# Object-Oriented Echo Perception and Cortical Representation in Echolocating Bats 

**DOI:** 10.1371/journal.pbio.0050100

**Published:** 2007-04-10

**Authors:** Uwe Firzlaff, Maike Schuchmann, Jan E Grunwald, Gerd Schuller, Lutz Wiegrebe

**Affiliations:** Department Biologie II, Ludwig-Maximilians-Universität München, Planegg-Martinsried, Germany; New York University, United States of America

## Abstract

Echolocating bats can identify three-dimensional objects exclusively through the analysis of acoustic echoes of their ultrasonic emissions. However, objects of the same structure can differ in size, and the auditory system must achieve a size-invariant, normalized object representation for reliable object recognition. This study describes both the behavioral classification and the cortical neural representation of echoes of complex virtual objects that vary in object size. In a phantom-target playback experiment, it is shown that the bat Phyllostomus discolor spontaneously classifies most scaled versions of objects according to trained standards. This psychophysical performance is reflected in the electrophysiological responses of a population of cortical units that showed an object-size invariant response (14/109 units, 13%). These units respond preferentially to echoes from objects in which echo duration (encoding object depth) and echo amplitude (encoding object surface area) co-varies in a meaningful manner. These results indicate that at the level of the bat's auditory cortex, an object-oriented rather than a stimulus-parameter–oriented representation of echoes is achieved.

## Author Summary

Bats can orientate and hunt for prey in complete darkness using echolocation. Bats use this extraordinary ability, not only to localize objects in space, but also to identify them. The same object, however, can come in different sizes. Here, we use a combination of psychophysical phantom-target experiments and electrophysiological recordings to investigate how echolocating bats perceive objects of different sizes, and how the echoes reflected from these objects are represented in the bat auditory cortex. We trained the neotropical bat Phyllostomus discolor to identify virtual objects, and found that these bats spontaneously associated scaled versions of these objects with the corresponding trained object. Interestingly, we identified neurons in the bat auditory cortex that respond to specific objects irrespective of object size. These findings highlight the sensory capabilities and sophisticated neural processes underlying bat echolocation. This suggests that like the visual system, echolocation meets an important requirement of an effective object-recognition system in that it allows the identification of objects independent of object size.

## Abstract 3

Auditory cortical neurons in echolocating bats fire selectively to objects despite changes in object size.

## Introduction

For both the visual and the auditory domain, the formation of perceptual objects from physical stimuli is an essential task. Reliable object recognition is complicated by the variability of naturally occurring objects, e.g., in object size. In the visual system, the effect of object size on object recognition and underlying neural substrates has been investigated in detail (for review see Logothetis and Sheinberg [1]). For example, neurons in the inferior temporal lobe can exhibit object-size invariant responses [2].

In the auditory domain, however, the definition of an object is not straightforward [3]. It is hypothesized that the auditory cortex segregates auditory objects depending on the auditory background, i.e., it adjusts its sensitivity for the boundaries of auditory objects along both the auditory time and frequency axes based on the spectrotemporal fluctuation statistics of the auditory background [4]. In humans, the analysis of auditory objects is thought to be implemented along “where” and “what” pathways in the auditory cortex [5,6], as recently corroborated in a combined functional magnetic resonance imaging (fMRI) and magneto encephalography (MEG) study [7]. The perception of size information in auditory objects, as part of the “what” analysis, has only recently been addressed. Human psychophysical studies have shown that information about speaker size is well preserved in human speech, that the human auditory system can segregate size information from information about the content, and thus that the auditory system can compensate for the effect of speaker size on perceived speech [8]: The same vowel pronounced by an adult and a child differs dramatically in its spectral content. However, it is readily perceived as the same vowel. Smith et al. [8,9] and Ives et al. [10] showed that recognition of vowels as well as the ability to judge relative size of speakers work well beyond the normally occurring range of speaker size. In an fMRI study, von Kriegstein et al. [11] showed that information about the vocal-tract length of a speaker, as an acoustic marker of body size, may be processed as early as the auditory thalamus and that an interaction between a voice's fundamental frequency (which can also mediate size information) may occur in non-primary auditory cortex.

For an echolocating bat, the definition of an auditory object is readily obtained: it is the information a bat gains from analyzing the echoes of self-generated sounds reflected by a physical, three-dimensional object in its environment. This clear definition of an echo-acoustic object allows the systematic investigation of the perception [12–14] and neural encoding of auditory object features [15,16] in an animal model. Behaviorally, echolocating bats can identify three-dimensional objects exclusively through the analysis of the echoes of their ultrasonic emissions. It was shown that bats use echolocation for object identification to find fruit [17,18] and flowers [19]. Schmidt et al. [20] showed that the gleaning bat Megaderma lyra uses echolocation to identify prey of different size. The bats also discriminated edible prey from dummies of the same size.

In all the above-mentioned cases, object normalization is crucial for compensating for inevitable variations in object size. In fact, it has been shown in behavioral experiments that bats are able to discriminate simple shapes independent of their size [21]. Acoustically, an object is defined by its impulse response (IR). The IR is the sum of the reflections when an object is ensonified with a Dirac impulse, i.e., an impulse of theoretically infinite shortness and amplitude. With increasing object size, there is a proportional change in both object surface area and object depth. Because the strength of the reflection depends on the size of the reflecting surface, the IR becomes louder with increasing object size. In addition, the IR becomes temporally expanded because object depth, and thus the temporal delay between single reflections, increases with increasing object size. This expansion corresponds to a compression of the spectral interference pattern. Taken together, much of the information about the three-dimensional structure of an object is represented in its IR, and much of the variability of the IR relates to the size of the object.

An echolocating bat does not perceive the IR as such, but it perceives the IR convolved with its echolocation call. Thus, the acoustic image of an object is imprinted on the echolocation call to produce an echo. The echo carries the acoustic properties of both the echolocation call and the IR. For a bat, it is essential to extract the IR from the echo [13,22]. Specifically, the loudness of an echo will not only depend on the loudness of the IR (encoding object size), but also on the loudness of the call. Echo loudness also depends on the distance between the bat and the object. Note, however, that the bat has full information about these parameters, because both the call loudness and the object distance are encoded in the auditory system. This information allows for a call loudness–independent and object distance–independent evaluation of the IR.

The aim of this study was to search for a size-invariant echo-acoustic object representation in a combined psychophysical and electrophysiological approach.

**Figure 1:**
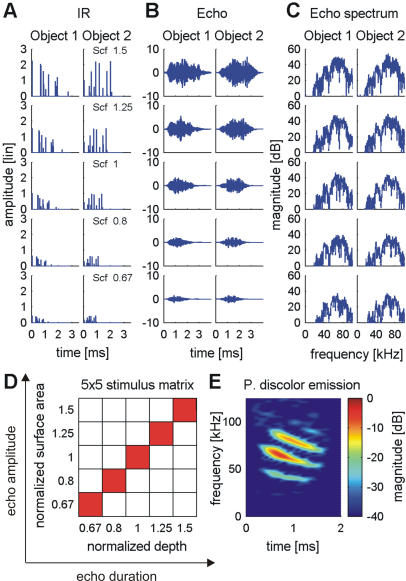
Stimuli Used for the Psychophysical and Electrophysiological Experiments (A) Impulse responses, (B) waveforms of the echo after convolution of an echolocation call with the IR, and (C) magnitude spectra of the echoes of object 1 and object 2 (left and right column, respectively). In the psychophysical experiments, the bats were trained to discriminate echoes of the standard objects shown the third row of A (scaling factor [Scf] 1). Once the bats had learned this task, presentations of scaled objects (scaling factors 0.67, 0.8, 1.25, and 1.5) were interspersed, and the spontaneous classification of these scaled objects was assessed. For the electrophysiological experiments, the IRs of the standard objects were scaled in terms of the delay and amplitude of the reflections with the same scaling factors and convolved with an echolocation call. The resulting 5 × 5 stimulus matrix is shown in (D). In this matrix the object-surface area and object depth vary along the vertical and the horizontal dimension, respectively. The red squares mark the properly scaled versions of the objects which are shown in (A–C). The physical parameters that changed in the vertical and horizontal dimension were amplitude and echo duration. Note, that all stimuli had very similar magnitude spectra. (E) Spectrogram of the echolocation call of P. discolor used for convolution with the IRs.

In the psychophysical phantom-object experiment, fruit-eating bats *(Phyllostomus discolor)* were trained to discriminate echoes of their sonar emissions. These echoes consisted of the emission convolved with the IRs of two objects. Each IR consisted of 12 randomly spaced reflections of different amplitude (Figure 1A, third row). Once the bats had learned this task, test trials were randomly interspersed, in which a scaled version of one of the standard objects was presented (Figure 1A), and the bats' spontaneous classification of theses scaled objects was assessed. The bats' psychophysical performance was compared to the performance of a spectrotemporal pattern recognizer being fed with auditory representations of the echoes, as they were perceived by the bats.

In the electrophysiological experiment, the ability of neurons in the auditory cortex of P. discolor to encode a normalized representation of the two objects that have been characterized psychophysically was tested. The IRs of both standard objects were scaled in terms of the delay and amplitude of the reflections and then convolved with a standard P. discolor echolocation call. For each object, the resulting stimuli are represented in a 5 × 5 matrix (Figure 1D) in which object depth and object surface area co-varied in a meaningful manner only along the diagonal axis, thus representing properly scaled versions of the object. Acoustically, the object–surface area parameter is encoded in the echo level, the object-depth parameter is encoded in the echo duration.

The psychophysical results show that P. discolor spontaneously classified most scaled versions of standard objects correctly. A population of cortical units was found that reflected normalized object features in their response rates. This population may serve as a substrate for the perceptual compensation of size-induced object variations.

## Results

### Psychophysics

**Figure 2:**
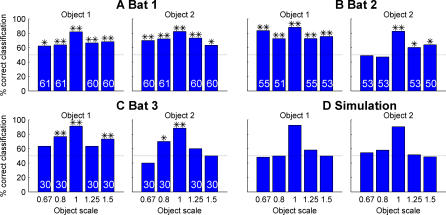
Psychophysical Classification Performance and Simulation Results Spontaneous classification of scaled virtual objects by three bats (A–C) and a simulation (D) based on a spectrotemporal pattern recognizer. Significantly correct classification of scaled objects is marked by a single star (*p* < 0.05) or two stars (*p* < 0.01). The number of test trials for each condition is superimposed on each bar. Although the spectrotemporal pattern recognizer cannot classify the scaled objects correctly, the bats' performance is in the majority of test conditions significantly better.

**Figure 3:**
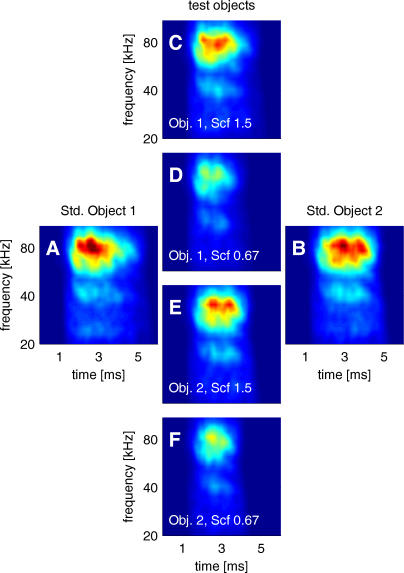
Auditory Spectrograms Generated by Echoes As They Are Perceived by the Bats Examples of auditory spectrograms produced by echoes generated with the IRs of both standard and scaled objects. The spectrograms incorporate the limits of spectral and temporal resolution of the auditory periphery of P. discolor. These spectrograms serve as inputs to the spectrotemporal pattern recognizer that tries to associate the spectrograms generated with different scaled objects (middle column [C–F]) to those generated with the two standard objects (A and B). Simulation results are shown in Figure 2D.

Behavioral results are based on a total of 4,500 trials obtained from three bats. Bat 1 spontaneously classified all four scaled versions for both of the two objects significantly correctly (Figure 2A). Bat 2 did so for all four scaled versions of object 1 and two of the scaled versions of object 2 (Figure 2B). Bat 3 showed a similar trend in evaluating the scaled objects, but failed to achieve significant performance in five of eight cases (Figure 2C). The performance of a spectrotemporal pattern recognizer is shown in Figure 2D. This pattern recognizer worked on a representation of the object echoes as generated by the bat's auditory periphery (see Materials and Methods, Figure 3). The mean-squared differences between the auditory spectrograms of the two standard objects (Figure 3A and 3B) and those of the scaled objects (examples in Figure 3C–3F) were calculated. Based on the mean-squared differences, the recognizer cannot reliably classify the peripheral representation of the scaled objects as one of the trained standard objects. Thus, even the bat that showed the weakest normalization behavior (bat 3) performed considerably better than the spectrotemporal pattern recognizer. Note that such a model could successfully predict responses in other echo-acoustic playback experiments [12,13]. These simulation results show that a dedicated neural mechanism beyond the auditory periphery is required to explain the bats' compensation for size-induced echo variations.

### Electrophysiology

**Figure 4:**
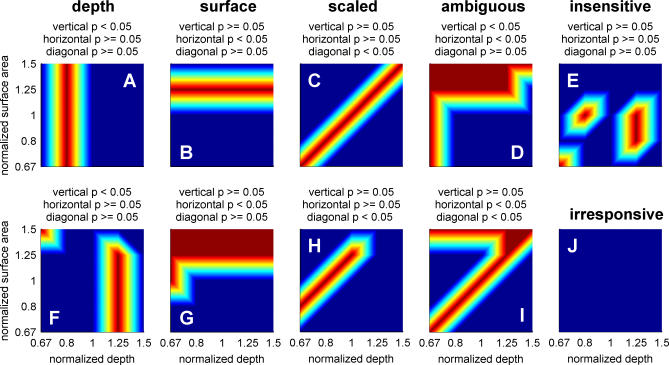
Illustration of the Principles Used to Categorize the Recorded Response Matrices The panels show synthetic response matrices and the results of the Kolmogorov-Smirnoff test applied along the vertical dimension (object depth), horizontal dimension (object surface area), and diagonal dimension (object scale). Test results are given above each response matrix. The results were used for the categorization as follows: if the null hypothesis could be rejected with *p* < 0.05 either for the vertical, horizontal, or the diagonal dimension, the matrix was assigned to the “depth” (A and F), “surface” (B and G), or “scaled” (C and H) category, respectively. If the null hypothesis could be rejected for more than one dimension, the matrix was categorized as “ambiguous” (D and I). If the null hypothesis could be rejected for none of the dimensions, the matrix was categorized as “insensitive” (E). The “irresponsive” category (J) consists of units that did not respond significantly, i.e., no analysis window could be set (see Materials and Methods). This precluded the use of the Kolmogorov-Smirnoff test for further analysis.

Recordings were derived from a total of 109 units from four bats (two females and two males, weighing between 33 and 44 g, lightly anaesthetized with a combination of medetomidin, midazolam, and fentanyl ([MMF]; see Materials and Methods). All units were tested with both objects. The stimuli for each object consisted of a standard echolocation call convolved with the 25 object IRs. For quantifying the neural responses, we computed the number of spikes for each of the 25 stimuli, with the spikes counted in a post-stimulus time window that was set separately for each unit according to the limits of statistically significant deviations from spontaneous activity (see Materials and Methods). The 25 responses to each object were arranged in a 5 × 5 matrix and normalized such that the maximum response was set to unity. Then, each response matrix was assigned to one of six categories (“scaled,” “depth,” “surface,” “ambiguous,” “insensitive,” and “irresponsive”; see Materials and Methods). An illustration of the categorization principles with synthetic response matrices is shown in Figure 4. Note that because each unit was tested with both objects, and units could respond differently to the two objects, percentages given below add up to more than 100%.

**Figure 5:**
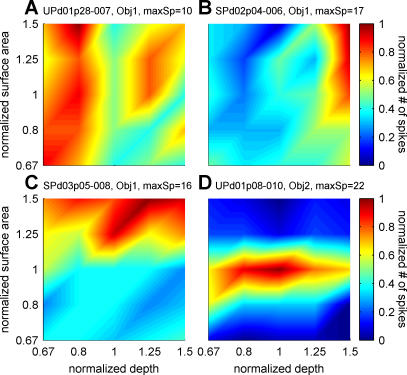
Responses of Cortical Units to Virtual Echo-Acoustic Objects (A and B) Normalized responses of units that responded best to a particular object depth (“depth” category). These units were largely insensitive to changes of object surface area. (C and D) Responses of units which encoded object surface area (“surface”-category). These units were largely insensitive to changes of object depth. The plots are arranged in the same way as the 5 × 5 stimulus matrix shown in Figure 1D. Abscissa: normalized object depth. Ordinate: normalized object surface area. MaxSp, maximum number of spikes; this number was taken as the divisor for the normalization of the responses.

Two recorded examples of response matrices assigned to the “depth” category are shown in Figure 5A and 5B. In these matrices, responses were strongest either to a particular object depth or to a combination of object depths, but were largely independent of object surface area. Such response matrices were recorded in 14/109 units (13%) for at least one of the two objects.

Two recorded examples of response matrices assigned to the “surface” category are shown in Figure 5C and 5D. In these matrices, responses were strongest either to a particular object surface area or to a combination of surface areas, but were largely independent of object depth. Such response matrices were recorded in 62/109 units (57%) for at least one of the two objects.

**Figure 6:**
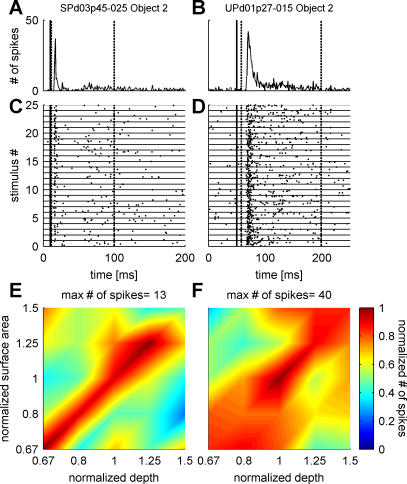
Examples of Two Cortical Units That Encoded Scaled Versions of Objects (A and B) Peri-stimulus, time histograms (PSTHs) summed up over all 25 stimuli for one object (ten repetitions for unit SPd03p45–025, 20 repetitions for unit UPd0127–015) (C and D) The corresponding raster plots. The stimulus is marked by the solid vertical line. Note that on this timescale, the stimulus duration, which ranged between 2.5 and 4 ms, corresponds to the width of the solid vertical line. The analysis window is indicated by the two dotted vertical lines in both the raster plot and the PSTH. (E and F) Normalized responses of the two units. Note that both units respond best to stimuli roughly along the diagonal axis of the 5 × 5 stimulus matrix shown in Figure 1D.

Most interestingly, in a third category, the response to stimuli along the diagonal axis, and thus to scaled versions of the stimuli, was strongest (“scaled”; Figure 6E and 6F). Such response matrices could be recorded in 14/109 units (13%) for at least one of the two objects. In these recordings, the response was not simply dependent on echo amplitude or duration, but on a meaningful combination of the two. The sophisticated properties of such a response matrix are reflected in the fact that the unit responded to this object equally strongly no matter whether it was small (the echo is faint and short) or large (the echo is loud and long). Thus, these units reflected normalized object features in their firing-rate. The effect is especially pronounced in the unit shown in Figure 6E. Except for the position in the upper right corner, stimuli along the diagonal axis of the 5 × 5 stimulus matrix evoked almost equally strong responses that clearly exceed the responses from other positions in the 5 × 5 matrix. In the other unit shown in Figure 6F, the strong responses are also evoked from positions neighboring the diagonal. However, the stimuli from positions in the lower right and upper left corner of the 5 × 5 matrix evoked only weak responses, so that the diagonal orientation of positions with strong responses still prevails. As can be seen from the raster plots and peri-stimulus time histograms (PSTH) in Figure 6A–6D, the stimuli evoked robust responses in both units.

A clear assignment to a distinct response category along one dimension (“depth,” “surface,” or “scaled”) was not always possible because some response matrices showed significant responses along more than one dimension. These matrices were categorized as “ambiguous” and were recorded in 10/109 units (9%) for at least one of the two objects**.** However, it is noteworthy that in four of these units, a significant response occurred also along the diagonal dimension either in response to object 1 or 2.

A considerable number of matrices did not show a significantly stronger response along any stimulus dimension and thus were categorized as “insensitive.” These matrices were recorded in 57/109 units (52%) for at least one of the two objects.

Finally, in some units, the response to the convolved echolocation calls was generally weak and did not reach significance (“irresponsive,” 8/109 units, 7%).

The number of units for which at least one of the recorded response matrices met the criteria for a response category are summarized in Table 1.

**Table 1:**
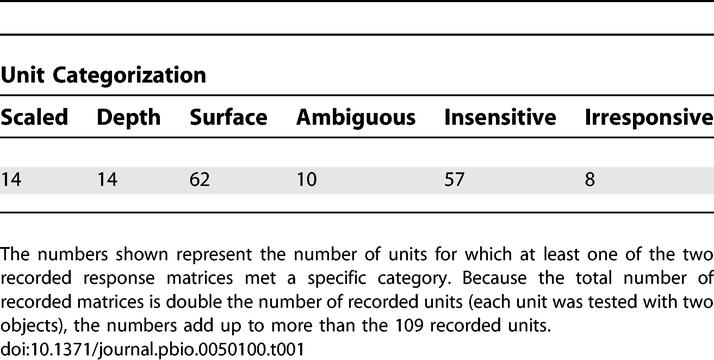
Categorization of the Recorded Units into Six Response Categories

Each unit was tested with two virtual objects. The combination of response categories in response to the two objects is given for each unit in Table 2. This table shows the number of units that, for the two objects, fall into a specific combination of response categories. In only 53/109 of the recorded units (49%), the recorded response matrices for the two objects were assigned to the same category. This is remarkable because the echoes generated with the IRs of both objects had very similar loudness and spectral content; they differed only in their temporal structure. Most units responded best to a particular object surface area (i.e., stimulus amplitude) or did not show any preference for a certain object dimension at all. A smaller number of units responded strongest to a particular object depth (i.e., stimulus duration) or to scaled versions of an object.

**Table 2:**
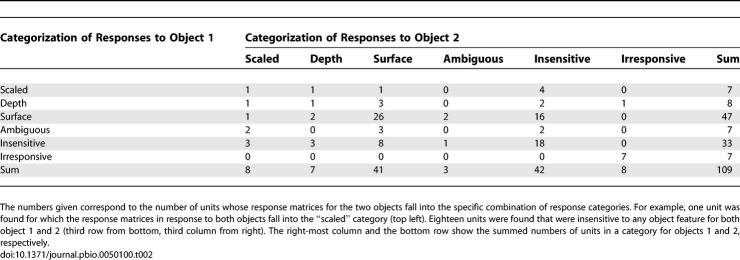
Observed Combinations of Response Categories for the Two Investigated Objects

Only one recorded unit was assigned to the “scaled” category in response to both objects. Most of the other units that responded in a “scaled” way to one object were “insensitive” to object depth, surface area, or scale in response to the other object.

### Spatial Distribution of Response Categories

**Figure 7:**
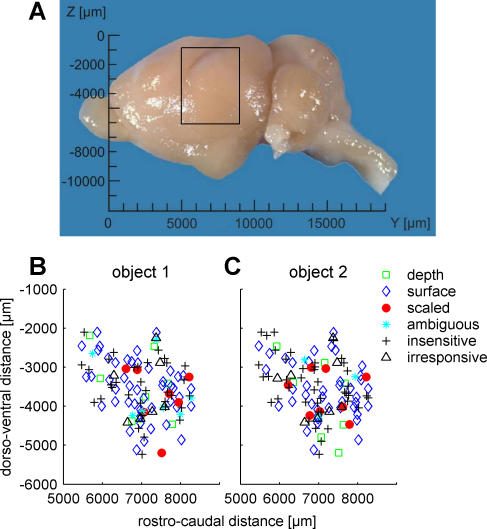
Location of Recording Sites in the Auditory Cortex of P. discolor The limits of the two scatter plots (B and C) are superimposed on the photograph of the P. discolor brain (A) (box). The scatter plots show the recording sites for units tested with objects 1 (B) and 2 (C). Units selective for scaled versions of an object (shown as filled red circles) were absent in the most anterior region of auditory cortex.

Recording sites were located at positions along the rostro-caudal axis over about 4,000 μm and along the dorsoventral axis over about 3,500 μm. This area roughly corresponds to the anatomically evaluated dimensions of the auditory cortex in P. discolor (S. Radtke-Schuller, personal communication). The different response categories were almost uniformly distributed over the whole cortical area from which recordings were derived, and no topological order could be detected (Figure 7B and 7C). However, units that responded most strongly to scaled versions of objects were absent in the most anterior region of auditory cortex. There is no detailed information published about different cortical fields in the auditory cortex of P. discolor. However, in the auditory cortex of a closely related species *(Carollia perspicillata),* dorsally located, non-tonotopic–organized fields have been distinguished from more ventrally located fields with tonotopic organization, presumably the primary auditory cortex and an anterior auditory field [23]. These data, combined with a preliminary partitioning of the P. discolor auditory cortex based on 563 units in our laboratory, suggest that scale-invariant response matrices were recorded in the high-frequency parts of the primary auditory cortex and the adjacent anterior auditory field. Notably, scale-invariant responses were absent in the non-tonotopical dorsal fields.

### Influence of Stimulus Level

**Figure 8:**
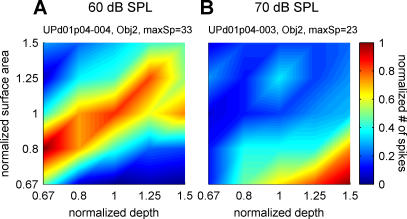
Influence of Overall Presentation Level on Responses of Cortical Units (A) The unit shown in the figure responded best to properly scaled versions of object 2 (diagonal axis) only at an adequate range of presentation levels. (B) A 10-dB change in the overall presentation level pushed the stimuli out of the range the unit was sensitive to. The overall presentation level is indicated above each panel. Abscissa: normalized object depth. Ordinate: normalized surface area. maxSp, maximum number of spikes.

The average presentation level for the recordings was set so that the loudest echoes were 20 to 30 dB above unit threshold. The surface-area axis of the presentation matrix translates to an echo-amplitude axis (larger surfaces produce louder echoes). Along the surface-area axis, echo level varies by 15 dB (see Neurophysiology, Materials and Methods). A change of the response category due to the change of stimulus level for either object 1 or object 2 occurred in 18 out of 21 units tested. The effect of changing the overall presentation level by 10 dB is illustrated in Figure 8. The data show that the categorization of a unit as “scaled” depends on the choice of an adequate range of presentation levels.

## Discussion

In a combined psychophysical and electrophysiological approach, this study tested the ability of the echolocating bat P. discolor to normalize for size-induced variations of virtual echo-acoustic objects. The psychophysical results showed that the bats spontaneously associated most scaled virtual objects with the corresponding standard object. A simulation of the psychophysical paradigm based on spectrotemporal pattern recognition indicates that a complex central auditory circuitry is required to explain the bats' performance. A neurophysiological correlate of this perceptual accomplishment is found in size-invariant responses to echoes from these virtual objects recorded from units in the auditory cortex. The population of these units comprised 13% of all cortical units investigated.

The psychophysical experiment was implemented as a real-time playback experiment. Consequently, any changes in the bat's echolocation call loudness, or in the distance between the bat and the virtual object in the setup, are preserved in the echo. If for example, a bat chose to emit a louder call towards a relatively faint IR from a small object, the perceived echo may be louder than the echo of a fainter call towards a louder IR. Thus, as in a natural echo-imaging task, this experimental paradigm requires the bats to evaluate the IR independent of the echo variations caused by call-loudness or object-distance variations. The psychophysical results show that, in most cases, the bats spontaneously normalized this extracted IR information for size-induced variations.

In the electrophysiological experiments, on the other hand, the animals were not echolocating, but were listening to the echoes passively. Thus, the neural circuitry had no information about both the virtual-object distance and the echolocation call loudness. Moreover, the 5 × 5 stimulus matrix for each of the two tested objects covered only a limited space along the dimensions of object depth and surface area. In consequence, the parameter space where a neuron potentially shows size-invariant responses could not always be covered by stimulating with a single 5 × 5 matrix. This is illustrated in Figure 8, where a variation of the overall presentation level adjusted the parameter space to fit the size-invariant response range. It is conceivable that in an actively echolocating animal, this range is adjusted according to the echolocation call loudness and the object distance.

**Figure 9:**
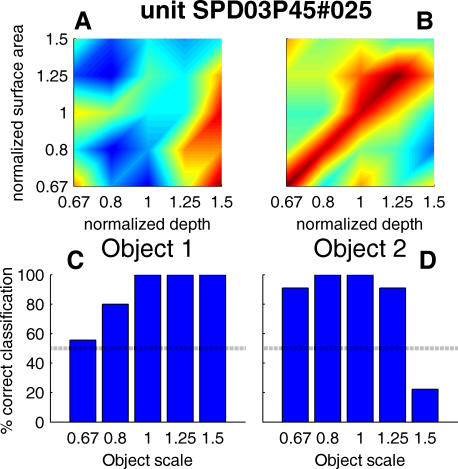
Putative Performance of Single Units in the Psychophysical Paradigm (A and B) show the rate responses of a unit to all versions of both standard objects. This unit responded best to scaled versions of object 2. Responses to object 1 were assigned to the “depth” category. (C and D) show the results of a simulation of both the behavioral discrimination and classification based on the rate responses of this cortical unit. The unit was not only able to respond best selectively to scaled versions of object 2, but also to discriminate the two objects and assign most responses to scaled versions of both objects to the correct standard.

The “scaled” category represents units that show the required response invariance along the object-size axis and may thus serve as a neural substrate for the bats' psychophysical tolerance to object scale. Reliable object recognition requires not only generality, i.e., response invariance to several objects belonging to the same class, but also specificity, i.e., different response characteristics for objects belonging to different object classes. The following analysis assessed to what extent neurons from the “scaled” category met both these requirements. This analysis was confined to those “scaled” category units that showed response invariance along the main diagonal (seven units) to allow a direct comparison to the behavioral results. The simulation paradigm is equal to that for the spectrotemporal pattern recognizer, i.e., the response strength to the “standard” echoes was compared to the response strengths to all test echoes. Simulation results with outputs from a “scaled” category unit as a simulation substrate instead of the spectrotemporal pattern recognizer are shown in Figure 9. This unit replicated the behavioral performance much better than the spectrotemporal pattern recognizer. Overall, two of the seven units combined both the required degree of specificity and generality in their responses, whereas the other five units did not meet the required object specificity.

In summary, a sizeable number of units showed response invariance along the object-size axis, but only two units combined this response invariance with the ability to discriminate between the two objects. In our view, both these levels of processing represent important stages towards a neural correlate of echo-acoustic object recognition. Compared to what is known about visual-object recognition, the current findings can only be seen as a first step towards an understanding of the neural basis of echo-acoustic object recognition.

### Comparison with Previous Studies

In nature, a bat can evaluate an object by integrating the echo information of the object from different ensonification angles. In fact, the echo spectra of bat-pollinated flowers changed significantly with the angle of incident sound, whereas echoes from single positions often were quite similar [19]. von Helversen [21] showed correct size-independent discrimination of real targets performed by the echolocating bat Glossophaga soricina. von Helversen proposed that correct object classification is guided by the changes in the spectral pattern of the returning echoes at different ensonification angles. Object recognition could then depend on serial integration of acoustic signals. The ability to integrate over a sequence of signal has been demonstrated in songbirds and mammals (e.g., see [24,25]). In contrast, the bats in the current study were confronted with the IRs of static, virtual objects. Thus, the bats received only a one-dimensional echo-acoustic image of these virtual objects. Nevertheless, the bats were able to classify scaled versions of these IRs correctly, although the information from different ensonification angles was missing. Hypothetically, the bats' echo-acoustic object-normalization ability will improve further when they can evaluate sequences of echoes from different ensonification angles.

### Cortical Representation of Objects

The stimuli used in this study differ fundamentally from those used in many previous investigations in which cortical sensitivity for a specific stimulus parameter is investigated. Namely, echoes from objects of different size differ both in echo intensity (larger objects produce louder echoes) and in the echo duration (larger objects produce longer echoes). Thus, a normalized object representation requires response invariance for a meaningful co-variation of these two acoustical parameters.

Heil [26] showed that neurons in the cat auditory cortex were tuned to the slope of the stimulus onset. In principle, such a mechanism could account for the described scale-invariant responses. When the size of the presented virtual objects changes, the change in signal duration affects the stimulus slope linearly. However, the corresponding change in surface area affects the stimulus slope quadratically (see Materials and Methods). Consequently, the slope of the stimulus onset is not constant over different scales of the same virtual object. Thus, tuning to onset slope cannot fully account for the described scale-invariant responses.

Galazyuk and Feng [27] showed that the best duration in duration-sensitive neurons in the auditory cortex of the little brown bat decreased with increasing amplitude. In our experiments, echo duration increased with object depth and thus co-varied in a systematic manner with echo amplitude for scaled versions of objects. Thus, stimulus intensity–dependent duration sensitivity of cortical units might be the underlying mechanism in units that encoded scaled versions of complex objects.

An important parameter influencing object normalization might be the bats own vocalization. It was shown for neurons in the inferior colliculus of the greater horseshoe bat *(Rhinolophus ferrumequinum)* that the bat's own vocalization altered the response to pure tones and frequency-modulated stimuli, presumably via direct neuronal influence of vocal activity onto collicular neurons [28]. This finding makes it highly likely that the response to scaled versions of acoustic objects is also influenced by the bat's own vocalization. As mentioned above, a mechanism like this would also be very favorable for bats, because the bats could compensate for differences in their outgoing echolocation calls that could otherwise be misattributed to object properties.

Units that responded most strongly to scaled versions of objects were not found in the most anterior parts of the auditory cortex, but were mostly located in the high-frequency parts of primary auditory cortex. This finding is interesting, because units that were sensitive to echo roughness were mainly located in anterior regions of the auditory cortex of P. discolor [15]. Because the processing of temporal envelope features is a prerequisite for encoding of echo roughness, the stimuli used in the present study provided no temporal envelope cues that could be used for object normalization; the spatial distribution of roughness-encoding units on the one hand and size-invariant units on the other hand seems to reflect a principle separation of encoding properties in anterior and posterior parts of the auditory cortex of P. discolor.

The current study supports the hypothesis that the auditory system has dedicated mechanisms to deal with the compensation of size-induced variations of acoustic sources. The fact that the size-invariant neural responses were obtained from individuals that had not been exposed to the stimuli behaviorally, indicates that this mechanism is not experience dependent, but hard wired.

## Materials and Methods

### Experimental animals.

The experimental animal used in this study was the New World bat Phyllostomus discolor (family: Phyllostomidae). The animals originated from a breeding colony in the Department Biologie II of the Ludwig-Maximilians-University in Munich. P. discolor emits short (<3 ms) broadband downward-modulated multiharmonic echolocation calls in the frequency range between 40 and 90 kHz (see Figure 1E). It feeds mainly on fruit, pollen, and insects [29], the insects being gleaned mainly from the vegetation.

### Psychophysics.

The psychophysical experiments were implemented as virtual-object playback experiments. The bats were required to evaluate echoes of their own echolocation calls. These echoes were generated by convolving in real time the calls with IRs of a virtual object. Thus, unlike in classical psychoacoustic experiments, the bats did not hear a sound unless they emitted echolocation calls.

Five adult P. discolor (four females, one male, body weight 30 to 40 g) took part in the training. The animals were housed in boxes (80 cm × 40 cm × 50 cm) with free access to water, and separated for sexes. In these boxes, they were only fed on days without training sessions, i.e., only for 2 d after a 5-d training period. During the training period, they were fed with banana pulp for reward. On the days without training, they had access to mealworms (larvae of Tenebrio molitor) ad libitum.

### Impulse responses.

A hundred IRs with 12 single reflections each of random relative level were generated (750 samples, 1.86 ms). Then, those two IRs with the largest mean-squared difference in the time domain were determined. These two IRs were taken as standard IRs of virtual objects 1 and 2.

Both IRs then were scaled with the following scaling factors (Scf): 0.67, 0.8, 1.25, and 1.5. The time delay of each reflection was scaled with Scf. The amplitude of each reflection was scaled with Scf^2^ because the level of a reflection is proportional to surface area and the latter is proportional to the square of the radius. Consequently, the IR produced with an Scf of 0.67 was 15 dB fainter than the same IR scaled with Scf of 1.5. The scaled IRs of both objects are shown in Figure 1A. All IRs had frequency-independent, white magnitude spectra. A convolution of these IRs with an echolocation call is equivalent to the multiplication of the spectra of call and IR. Thus, the high similarity of the echo spectra shown in Figure 1C reflects the similarity of the IR spectra. This similarity occurs because the 12 reflections with random delays do not produce a systematic spectral ripple.

### Experimental set up.

The bats were trained in a two-alternative, forced-choice (2-AFC) playback setup as used in former experiments with P. discolor [12,15,30]. It consists of a Y-shaped maze, inversely mounted on the wall of an echo-attenuated chamber at an angle of 45°. A starting perch was located at the top end, and a feeder was mounted at the end of each leg. The angle between the legs was 60°. A 1/4-in microphone (Microtech Gefell MK 301; Gefell, Germany) was located in the middle of the maze to pick up the bats' sonar emissions. The ultrasonic emissions were amplified (model 2160; Bruel & Kjaer, Naerum, Denmark) and then digitized by a data-acquisition board (data acquisition processor 5200a; Microstar, Bellevue, Washington, United States) at a sampling rate of 250 kHz. On the processor, a software trigger was implemented to look for input values larger than about a tenth of the possible input range. When triggering occurred, 500 samples (50 before the trigger event, and 450 after the event) were processed. The data-acquisition board convolved this input with the desired IR by zero padding both the recorded call and the IR to 2,048 samples, and multiplying the complex spectra of the recorded call and the IR. The resulting artificial echo was then again amplified (model 6110; Harman/Kardon, Château du Loir, France) and played back via an ultrasonic speaker (model EAS10 TH800D; Matsushita, Osaka, Japan) which was placed at a distance of 20 cm from the starting position in the middle between the two legs. The digital processing time for the echo generation was 6 ms. Together with the physical delay of the sound from the bat to the microphone and from the speaker to the bat, this resulted in an overall echo delay of about 7.5 ms. The corresponding virtual-object distance was 127 cm. This distance was fixed for both objects and scaling factors. The target strength of the virtual objects depended on the IR and ranged between −21 and −6 dB. The experimenter was seated outside the chamber, controlling the experimental procedure via a computer interface and an infrared camera. Data acquisition and analysis was implemented in Matlab 6.5 (Mathworks, Natick, Massachusetts, United States).

### Training procedure.

In a 2-AFC experiment with food reward, five individuals of P. discolor were trained to discriminate the two standard IRs, representing two virtual objects. Dependent on the presented object, the bat had to crawl into leg one (object 1) or two (object 2) to obtain a food reward. Three out of five trained animals were able to solve this task. When the bats' performance in these standard trials exceeded 80% correct, test trials were randomly interspersed with a probability of 25%. In these test trials, scaled versions of object 1 or 2 were presented. Test trials were always rewarded, independent of the bats' decision. Thus, in the test trials, the bats' spontaneous classification of the scaled objects as either object 1 or object 2 was assessed. Data acquisition stopped when the slowest animal had performed at least 30 trials for each scaled object. The spontaneous performance for each scaled object was numerically tested for significance by simulating 10,000 repetitions of the 2-AFC experiment with the given number of trials and a random performance. Significance was set at *p* < 0.05.

### Simulation of the classification of scaled virtual objects via auditory spectrograms in *P. discolor.*

A bat does not hear the IR of an object itself, but the IR convolved with its echolocation call. Moreover, these convolved calls are modified in the auditory periphery of the bats. To simulate the classification of the scaled objects used in this study, we have to take account of these modifications. We simulated the auditory periphery up to the stage of the auditory nerve. First, we convolved each IR of each object with a standard echolocation call. Then, the convolved signal was sent through outer and middle ear filters that mimicked the absolute thresholds of P. discolor as described by Esser and Daucher [31]. To simulate the inner ear characteristics, we applied a gammatone filter bank, consisting of 25 channels with center frequencies equally spaced on a log frequency axis between 20 and 110 kHz. The transfer function of the gammatone filters is designed to mimic the shape of the distortion product, otoacoustic-emission tuning curves of P. discolor as measured by Wittekindt et al. [32]. After half-wave rectification and exponential compression, we applied a phase-locking filter (cutoff frequency: 1 kHz; 12 dB/octave). The resulting signal is then resampled at 20 kHz. Thus, we obtained the auditory spectrograms generated by echoes as they are perceived by the bats in the experimental setup.

The auditory spectrograms generated with the two standard objects are shown in Figure 3A and 3B. Examples of auditory spectrograms for four of the eight scaled versions of the standard objects are shown in Figure 3C–3F.

Next, the mean-squared differences between the auditory spectrograms generated with scaled objects and those generated with standard objects were calculated. Based on these mean-squared differences, the simulation classified the auditory spectrograms of the scaled objects as either similar to the spectrograms of standard object 1 or standard object 2. Thus, the simulation works as a spectrotemporal pattern recognizer. Note that this simulation has no concept of scaling, and thus it serves as a null hypothesis for a behavioral test of echo-acoustic object normalization.

### Neurophysiology—surgery.

Four adult bats were used for the neurophysiological experiments. All experiments complied with the principles of laboratory animal care and were conducted under the regulations of the current version of the German Law on Animal Protection (approval 209.1/211-2531–68/03, Reg. Oberbayern). The principle surgical procedure has been described in detail elsewhere [33]. In brief, bats were anesthetized using MMF (medetomidin 0.4 μg, midazolam 4 μg, and fentanyl 0.04 μg per gram body weight). The skin overlying the skull was opened along the midline and the skull surface was freed from tissue. A small metal tube was fixed to the skull using a microglass composite in order to fixate the animal to a stereotaxic device, and the accurate skull position in stereotaxic coordinates was determined as described in detail elsewhere [34].

### Neurophysiology—stimulus production and recording of neural responses.

Experiments were conducted in a sound-attenuated chamber. Acoustic stimuli were computer generated (Matlab; Mathworks), digital-analog converted (RX6 [Tucker Davis Technologies, Gainesville, Florida, United States], sampling rate: 260 kHz), attenuated (PA5; Tucker Davis Technologies), and binaurally presented via ultrasonic earphones with a flat frequency response (±3 dB) between 10 and 100 kHz [35].

Stimuli consisted of a typical echolocation call of P. discolor (Figure 1E) convolved with IRs identical to the IRs used in the psychophysical experiments. The standard IRs of object 1 and object 2 were scaled in terms of the delay and amplitude of the reflections by scaling factors of 0.67, 0.83, 1, 1.25, and 1.5. The resulting stimuli can be represented in a 5 × 5 matrix in which object surface area (vertical dimension) and object depth (horizontal dimension) co-varied in a meaningful manner only along the diagonal axis, thus representing properly scaled versions of the object (Figure 1D). Along the vertical dimension, the acoustical parameter that changed was amplitude, whereas along the horizontal dimension, echo duration changed (Figure 1D). For both virtual objects, the echoes span a level range of 15 dB. Dependent on the duration of the IRs, the resulting stimulus duration varied between about 2.5 and 4 ms (Figure 1B). Note that all 50 echoes from the two objects had very similar spectral envelopes (Figure 1C).

The sound level was chosen so that the loudest echoes were 20 to 30 dB above a unit's pure-tone threshold. The set of 50 echoes was presented in a randomized order at a repetition period of 770 ms (inter-stimulus interval between 766 and 767.5 ms). Within each period, the echo was preceded by a 10 to 50 ms silent period to determine the spontaneous activity (see below). The set of 50 echoes was presented 10 or 20 times.

For electrophysiological recordings, bats were anaesthetized with MMF (0.4 μg, 4 μg, and 0.04 μg per gram body weight, respectively). During recording, anesthesia was maintained by injecting the half of the initial dose of MMF every 2 h. Recording sessions could last up to 5 h per day and were repeated 4 d a week. Action potentials from neurons in the auditory cortex were recorded extracellularly using either glass microelectrodes filled with 2 M NaCl and 4% Pontamine Sky Blue (3–8 MΩ impedance) or carbon fiber microelectrodes (Carbostar-1 [Kation Scientific, Minneapolis, Minnesota, United States], 0.4–0.8 MΩ impedance). Because it was not always possible to clearly discriminate the activity of a single neuron, the term *unit* will be used in the following to describe the activity of one neuron to clusters of three neurons recorded at a distinct recording site. Neural activity was monitored audiovisually, and threshold and best frequency of a unit were roughly determined. Action potentials were amplified using conventional methods and recorded using an AD converter (RP2.1 [Tucker Davis Technologies], sampling rate: 25 kHz) and Brainware (Tucker Davis Technologies). Electrode penetrations were made tangentially to the brain surface. After the completion of an experiment, lesions were made to the brain in order to reconstruct the position of recording sites from subsequent histological processing in standardized coordinates of a brain atlas of P. discolor (A. Nixdorf, T. Fenzl, B. Schwellnus, unpublished data).

### Data analysis.

Spike responses from all 25 stimuli for each object were displayed as raster plots (see Figure 6C and 6D). An analysis window was set automatically by moving a 10-ms window in 1-ms steps over the time course of recorded activity and computing a Wilcoxon signed rank test (*p* < 0.01, Matlab Statistics Toolbox, Mathworks) over the 25 stimuli and the first 10–50 ms preceding each stimulus (spontaneous activity). The first point at which two successive windows led to significant responses was taken as the start of the analysis window; the last position of two successive significant windows was taken as the end of the analysis window. Spikes were summed in the analysis window and normalized such that the maximum number of spikes was set to unity. The normalized responses were arranged as color-coded plots in a 5 × 5 matrix corresponding to the stimulus matrix (cf. Figure 1D). If no significant response was detected by the analysis window, the neuron was categorized as “irresponsive” (Figure 4J).

In all other cases, a Kolmogorov-Smirnov test (kstest2, Matlab Statistics Toolbox, Mathworks) was used to test whether the five responses, e.g., for a specific object depth, belonged to the same, continuous distribution as all other responses in the response matrix. Exemplary, synthetic response matrices, in which this hypothesis can be rejected with *p* < 0.05, are illustrated in Figure 4A and 4F. This analysis was performed, not only along the vertical dimension (object depth), but also along the horizontal dimension (object surface area, Figure 4B and 4G) and along the diagonal dimension (object scale, Figure 4C and 4H). The test along the diagonal was performed for the main diagonal and one parallel above and below. This was done in order to avoid miscategorizing units due to the comparison of only low numbers of positions in the 5 × 5 matrix (the shortest diagonal line in the matrix would contain only two positions). If the null hypothesis could be rejected for more than one of the three tested dimensions, the response matrix was categorized as “ambiguous.” If the null hypothesis could be rejected for none of the three dimensions, the response matrix was categorized as “insensitive.”

## Abbreviations

IR: impulse response
MMF: medetomidin, midazolam, and fentanyl
Scf: scaling factor

## References

[pbio-0050100-b001] Logothetis NK, Sheinberg DL (1996). Visual object recognition. Annu Rev Neurosci.

[pbio-0050100-b002] Ito M, Tamura H, Fujita I, Tanaka K (1995). Size and position invariance of neuronal responses in monkey inferotemporal cortex. J Neurophysiol.

[pbio-0050100-b003] Griffiths TD, Warren JD (2004). What is an auditory object?. Nat Rev Neurosci.

[pbio-0050100-b004] Nelken I (2004). Processing of complex stimuli and natural scenes in the auditory cortex. Curr Opin Neurobiol.

[pbio-0050100-b005] Rauschecker JP, Tian B (2000). Mechanisms and streams for processing of “what” and “where” in auditory cortex. Proc Natl Acad Sci U S A.

[pbio-0050100-b006] Rauschecker JP (1998). Parallel processing in the auditory cortex of primates. Audiol Neurootol.

[pbio-0050100-b007] Ahveninen J, Jaaskelainen IP, Raij T, Bonmassar G, Devore S (2006). Task-modulated “what” and “where” pathways in human auditory cortex. Proc Natl Acad Sci U S A.

[pbio-0050100-b008] Smith DR, Patterson RD, Turner R, Kawahara H, Irino T (2005). The processing and perception of size information in speech sounds. J Acoust Soc Am.

[pbio-0050100-b009] Smith DR, Patterson RD (2005). The interaction of glottal-pulse rate and vocal-tract length in judgements of speaker size, sex, and age. J Acoust Soc Am.

[pbio-0050100-b010] Ives DT, Smith DR, Patterson RD (2005). Discrimination of speaker size from syllable phrases. J Acoust Soc Am.

[pbio-0050100-b011] von Kriegstein K, Warren JD, Ives DT, Patterson RD, Griffiths TD (2006). Processing the acoustic effect of size in speech sounds. Neuroimage.

[pbio-0050100-b012] Grunwald JE, Schörnich S, Wiegrebe L (2004). Classification of natural textures in echolocation. Proc Natl Acad Sci U S A.

[pbio-0050100-b013] Weissenbacher P, Wiegrebe L (2003). Classification of virtual objects in the echolocating bat, Megaderma lyra. Behav Neurosci.

[pbio-0050100-b014] Simmons JA, Neretti N, Intrator N, Altes RA, Ferragamo MJ (2004). Delay accuracy in bat sonar is related to the reciprocal of normalized echo bandwidth, or Q. Proc Natl Acad Sci U S A.

[pbio-0050100-b015] Firzlaff U, Schörnich S, Hoffmann S, Schuller G, Wiegrebe L (2006). A neural correlate of stochastic echo imaging. J Neurosci.

[pbio-0050100-b016] Sanderson MI, Simmons JA (2002). Selectivity for echo spectral interference and delay in the auditory cortex of the big brown bat Eptesicus fuscus. J Neurophysiol.

[pbio-0050100-b017] Kalko EKV, Condon MA (1998). Echolocation, olfaction and fruit display: How bats find fruit of flagellichorous cucurbits. Funct Ecol.

[pbio-0050100-b018] Thies W, Kalko EKV, Schnitzler HU (1998). The roles of echolocation and olfaction in two Neotropical fruit-eating bats, Carollia perspicillata and *C. castanea,* feeding on Piper. Behav Ecol Sociobiol.

[pbio-0050100-b019] von Helversen D, von Helversen O (2003). Object recognition by echolocation: A nectar-feeding bat exploiting the flowers of a rain forest vine. J Comp Physiol [A].

[pbio-0050100-b020] Schmidt S, Hanke S, Pillat J (2000). The role of echolocation in the hunting of terrestrial prey—New evidence for an underestimated strategy in the gleaning bat, Megaderma lyra. J Comp Physiol [A].

[pbio-0050100-b021] von Helversen D (2004). Object classification by echolocation in nectar feeding bats: Size-independent generalization of shape. J Comp Physiol [A].

[pbio-0050100-b022] Simmons JA (1989). A view of the world through the bat's ear: The formation of acoustic images in echolocation. Cognition.

[pbio-0050100-b023] Esser KH, Eiermann A (1999). Tonotopic organization and parcellation of auditory cortex in the FM-bat Carollia perspicillata. Eur J Neurosci.

[pbio-0050100-b024] Hulse SH, Cynx J (1986). Interval and contour in serial pitch perception by a passerine bird, the European starling *(Sturnus vulgaris)*. J Comp Psychol.

[pbio-0050100-b025] Ralston JV, Herman LM (1995). Perception and generalization of frequency contours by a bottle-nosed dolphin *(Tursiops truncatus)*. J Comp Psychol.

[pbio-0050100-b026] Heil P (1997). Auditory cortical onset responses revisited. II. Response strength. J Neurophysiol.

[pbio-0050100-b027] Galazyuk AV, Feng AS (1997). Encoding of sound duration by neurons in the auditory cortex of the little brown bat, Myotis lucifugus. J Comp Physiol [A].

[pbio-0050100-b028] Schuller G (1979). Vocalization influences auditory processing in collicular neurons of the CF-FM bat, Rhinolophus ferrumequinum. J Comp Physiol [A].

[pbio-0050100-b029] Novak RM (1994). Walker's bats of the world.

[pbio-0050100-b030] Schuchmann M, Hübner M, Wiegrebe L (2006). The absence of spatial echo suppression in the echolocating bats Megaderma lyra and Phyllostomus discolor. J Exp Biol.

[pbio-0050100-b031] Esser KH, Daucher A (1996). Hearing in the FM-bat *Phyllostomus discolor:* A behavioral audiogram. J Comp Physiol [A].

[pbio-0050100-b032] Wittekindt A, Drexl M, Kössl M (2005). Cochlear sensitivity in the lesser spear-nosed bat, Phyllostomus discolor. J Comp Physiol [A].

[pbio-0050100-b033] Schuller G, O'Neill WE, Radtke-Schuller S (1991). Fascilitation and delay sensitivity of auditory cortex neurons in CF-FM bats Rhinolophus rouxi and *Pteronotus p. parnellii*. Eur J Neurosci.

[pbio-0050100-b034] Schuller G, Radtke-Schuller S, Betz M (1986). A stereotaxic method for small animals using experimentally determined reference profiles. J Neurosci Methods.

[pbio-0050100-b035] Schuller G (1997). A cheap earphone for small animals with good frequency response in the ultrasonic frequency range. J Neurosci Methods.

